# Spectroscopic Analysis of Melatonin in the Terahertz Frequency Range

**DOI:** 10.3390/s18124098

**Published:** 2018-11-23

**Authors:** Uroš Puc, Andreja Abina, Anton Jeglič, Aleksander Zidanšek, Irmantas Kašalynas, Rimvydas Venckevičius, Gintaras Valušis

**Affiliations:** 1 Jožef Stefan International Postgraduate School, Ljubljana 1000, Slovenia; andreja.abina@mps.si (A.A.); anton.jeglic@gmail.com (A.J.); aleksander.zidansek@mps.si (A.Z.); 2 Institute of Computational Physics, School of Engineering, Zurich University of Applied Sciences (ZHAW), Technikumstrasse 9, Winterthur 8401, Switzerland; 3 Department of Condensed Matter Physics, Jožef Stefan Institute, Ljubljana 1000, Slovenia; 4 Faculty of Natural Sciences and Mathematics, University of Maribor, Maribor 2000, Slovenia; 5 Optoelectronics Department, Center for Physical Sciences and Technology, Vilnius 01108, Lithuania; irmantak@ktl.mii.lt (I.K.); r.venckevicius@gmail.com (R.V.); gintaras.valusis@ftmc.lt (G.V.); 6 Danske Bank, Saltoniškių g. 2, Vilnius 08500, Lithuania

**Keywords:** hormones, terahertz spectroscopy, terahertz imaging, melatonin, active pharmaceutical ingredient, Fourier transform infrared spectroscopy, pharmaceutical industry

## Abstract

There is a need for fast and reliable quality and authenticity control tools of pharmaceutical ingredients. Among others, hormone containing drugs and foods are subject to scrutiny. In this study, terahertz (THz) spectroscopy and THz imaging are applied for the first time to analyze melatonin and its pharmaceutical product Circadin. Melatonin is a hormone found naturally in the human body, which is responsible for the regulation of sleep-wake cycles. In the THz frequency region between 1.5 THz and 4.5 THz, characteristic melatonin spectral features at 3.21 THz, and a weaker one at 4.20 THz, are observed allowing for a quantitative analysis within the final products. Spectroscopic THz imaging of different concentrations of Circadin and melatonin as an active pharmaceutical ingredient in prepared pellets is also performed, which permits spatial recognition of these different substances. These results indicate that THz spectroscopy and imaging can be an indispensable tool, complementing Raman and Fourier transform infrared spectroscopies, in order to provide quality control of dietary supplements and other pharmaceutical products.

## 1. Introduction

Hormones are crucial for the control and regulation of many different human body processes and everyday life activities. Hormone-based drugs and their synthetic analogous produced from natural sources, or by total synthesis, represent an important group of drug materials [1]. Novel rapid and accurate analytical methods for the determination of hormones in biological samples, pharmaceutical products, water and food, and to ensure product quality control are needed [2,3]. Several spectroscopic methods including UV-VIS spectroscopy [4], nuclear magnetic resonance (NMR) spectroscopy [5], infrared spectroscopy (IR) [6,7], and Raman spectroscopy [8,9] have been successfully applied in the chemical analysis of hormone-based drugs. Among the IR-based methods, the emphasis has been given to Fourier transform infrared (FTIR) spectroscopy and near-infrared (NIR) spectroscopy [9,10,11]. Developments in the terahertz (THz) technology show a promising potential for applications in many fields [12,13,14,15,16,17,18] with great emphasis on the pharmaceutical industry [19,20,21,22,23], as the far-infrared frequency range may be employed for the chemical screening and analysis of substances. Terahertz waves lie between the microwave and infrared region of the electromagnetic spectrum. Spectral responses in THz frequency range contain information of intra- and inter-molecular motions, and thus reflect a single molecular structure and molecular arrangements [8]. THz waves can penetrate through many materials, have low photon energy that does not damage the samples, and the waves are coherent, which gives an advantage over Raman and FTIR spectroscopies. As the THz time-domain spectroscopy is coherent, it displays much higher sensitivity and dynamic range in comparison to FTIR [24]. Furthermore, the combination of these different techniques can provide a deeper understanding of a sample’s structure.

Thus far, THz spectroscopy has been applied to study polymorphism [25,26], cocrystals [27], crystallinity [28], hydrates [29], hydrogen-bonding [30], helical antennae related effects [31], and conformational disorder [32] of pharmaceutical compounds. Furthermore, an additional modality of systems allowing to perform THz imaging provides a visualization of individual chemical compounds within heterogeneous samples, such as pharmaceutical pellets. A THz imaging analytical method, known as component spatial pattern analysis [33], has been employed for chemical mapping of cocrystals [34] and for the determination of coating layer thickness and density [35]. Since plastics are almost transparent to THz waves, THz spectroscopic imaging can also reveal the distribution of both active pharmaceutical ingredient (API) and other important constituents within the pharmaceutical pellets covered by blisters. Some research on hormone-based drugs has already been performed using THz spectroscopy, including studies of the vibrations of corticosteroids [8,36,37], the structure of molecules in crystals of steroid hormones [38,39], and the conformation of naphthols to mimic natural hormones with estrogenic-like activity [40] where they were able to detect hormones in blood [41] for diagnostics of diabetes. THz spectroscopic experiments with steroid hormones including testosterone, estradiol, and estriol were also performed comparing the obtained results with Raman spectra [8,36,39,42].

In this paper, we demonstrate the application of THz spectroscopy and spectroscopic THz imaging to study melatonin and its pharmaceutical product. Melatonin is one of the most studied biological substances in the last fifty years [43]. It is a naturally occurring hormone belonging to tryptamines and is mainly produced and released by the pineal gland, which is of great importance in the regulation of various human body physiological functions [44]. Today, melatonin is available in two formulations, as an immediate-release dietary supplement and a prolonged-release prescription-only pharmaceutical drug. Recent regulations by the Food and Drug Administration (FDA) emphasize the importance of quality control and authentications of dietary supplements such as melatonin. Fast and reliable detection methods of melatonin in final products are needed in order to eliminate counterfeit or poor quality products before they reach end users. To the best of our knowledge, THz spectroscopy and THz imaging studies of melatonin and its pharmaceutical product have not been reported yet. FTIR spectrum studies of melatonin close to THz frequency range [10] have been performed, whereby infrared absorption spectra of melatonin in 1700–70 cm^−1^ were measured. The experimental FTIR spectrum of melatonin showed one characteristic absorption peak below 137 cm^−1^, corresponding to 4.11 THz. In Raman spectrum of melatonin two peaks were observed [10], one around 145 cm^−1^ (4.35 THz) and another around 120 cm^−1^ (3.60 THz). 

Here, we present an experimental study of THz absorption spectra of pure melatonin and its pharmaceutical product Circadin by applying several techniques in the THz frequency range—FTIR, coherent THz time-domain spectroscopy (THz-TDS), and spectroscopic THz imaging. The usefulness of THz spectroscopic methods for qualitative and quantitative analysis of melatonin and its final drug product are demonstrated. Our work shows that THz spectroscopy can provide a complementary analysis tool in addition to Raman and FTIR spectroscopy, to characterize hormone-based prescription drugs and food supplements available to a wide group of end users.

This article is organized as follows. Section 2 provides the materials selection and sample preparation, followed by Section 3, in which the methods are described and illustrated. Section 4 summarizes and discusses the results obtained by the measurements.

## 2. Materials

**Figure 1:**
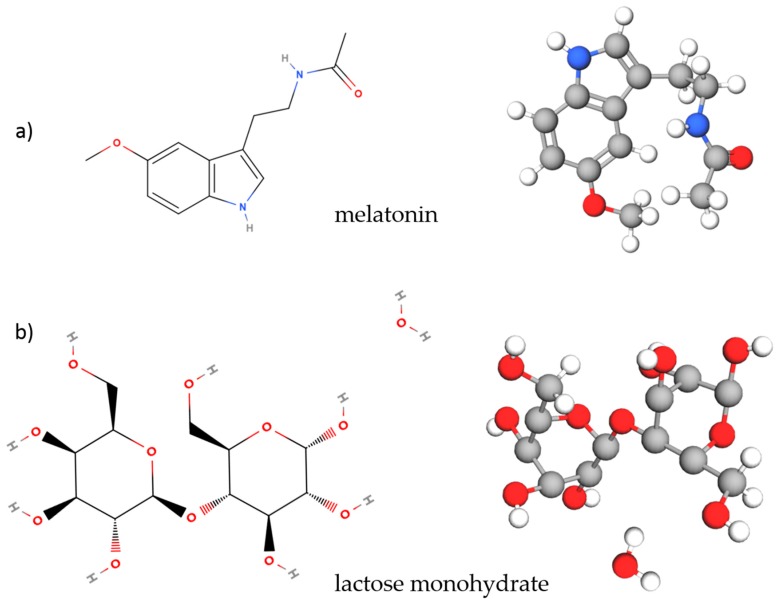
Structural formula and 3D model of (**a**) melatonin and (**b**) lactose monohydrate.

Melatonin (N-Acetyl-5-methoxytryptamine) with purity greater than 98% and polyethylene powder (PE) with an average particle size of 53–75 µm were purchased from Sigma-Aldrich and used as obtained. Circadin prolonged-release tablets (2 mg) were also obtained, produced by Neurim Pharmaceuticals. Each Circadin tablet contains 2 mg of melatonin and 80 mg of lactose monohydrate (see structural formula and molecular 3D models in Figure 1), as well as other ingredients: Ammonio methacrylate copolymer type B, calcium hydrogen phosphate dihydrate, silica, talc, and magnesium stearate. 

PE was selected as a reference because of its negligible absorption at the THz frequencies. Circadin pellets were first crushed to a fine powder in a mortar and pestle. All powder samples were then dried for 24 h at 65 °C in an oven to reduce water content. For FTIR and THz measurements, we prepared sample pellets by diluting the powdered materials in a reference PE material. The selected powders for pellet compression were also mixed and grinded by pestle in a mortar. Experimental samples in the form of pellets with a diameter of 18.4 mm were prepared by applying pressure of 18.9 kN/cm^2^ for 50 s at room temperature, using a manual hydraulic press. All samples had the same mass of 320 mg and thickness of 1.5–1.7 mm. The reference PE pellet consisted of 100 wt% PE. Melatonin pellets were prepared with a concentration of 5 wt% and 10 wt%, and Circadin pellets with 5 wt% and 10 wt% of the substance, whereas the rest of the mixture in pellets was PE powder. The composition of different pellets used for THz measurements is summarized in Table 1.

## 3. Methods

**Figure 2:**
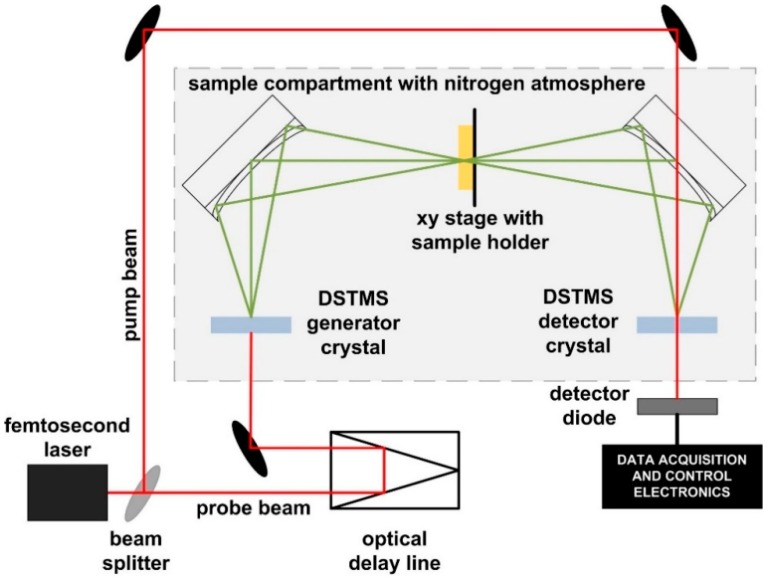
Schematic drawing of the THz spectroscopy and imaging system based on DSTMS organic crystals in transmission geometry.

An in-house modified TeraIMAGE system by Rainbow Photonics AG, Zurich Switzerland, was used for spectroscopic measurements as shown in Figure 2. It allows sample investigation in transmission geometry from 1.5 THz to 6 THz. In this experiment, the measurements were performed up to 4.5 THz, since from this frequency onwards the signal-to-noise ratio was too low due to the high absorption of selected samples. The system is based on a femtosecond erbium-doped laser (Menlo Systems) with an average output power greater than 150 mW, pulse duration of less than 90 fs, and a repetition rate of 100 MHz. In this setup, the pump beam hits the DSTMS (4-N,N-dimethylamino-4’-N’-methyl-stilbazolium-2,4,6-trimethylbenzenesulfonate) nonlinear electrooptic organic crystal [45] which generates THz waves by employing the optical rectification principle. The generated THz waves are guided by elliptic mirrors through the sample compartment to the DSTMS detector crystal which detects THz waves by the THz-induced lensing detection principle [46]. A detailed description of the apparatus is described elsewhere [15]. Due to a strong absorption of THz waves in water vapors, the sample compartment was purged with nitrogen gas. Absorbance was calculated as −log(*I/I_0_*), where the terms *I* and *I_0_* denote the THz beam intensity measured in the presence and absence of a sample in the sample compartment, respectively. The THz imaging system was based on the same TeraIMAGE in-house modified system with an addition of a translation stage in the sample compartment. It allows for a raster-scan imaging of samples under the investigation. Images were acquired with a resolution of 1 mm by raster scanning an area of 44 mm × 27 mm. During the scan, the sample compartment was continuously purged with the nitrogen gas to minimize the effects of water absorption. The spatial resolution of our THz-TDS imaging system is specified by the manufacturer, and can achieve 100 µm. The spatial resolution here is mainly limited by the THz diffraction limit. To overcome this limit, different approaches in improving the resolution to sub-THz wavelengths were proposed, e.g., using near-field techniques, generating the THz signal at the surface of the investigated sample, detection of THz by very small probes, computational imaging methods, etc. [47,48,49]. By using these techniques, spatial resolutions in nanometer range were achieved. However, due to practical reasons in THz-TDS spectroscopic raster scanning whereby each pixel acquisition could take several minutes due to the slow mechanical delay-line operation, a spatial resolution of 1 mm was chosen here as it provides sufficient information for the intended aim of this study.

To record Fourier spectra, a home-developed FTIR spectrometer with a vacuum option was used for measurements in the THz frequency range. The THz transmission spectra *I/I_0_* and absorbance spectra −log(*I/I_0_*) were measured with the resolution of 1 cm^−1^. The terms *I* and *I_0_* denote the THz beam intensity measured in the presence and absence of a sample in the sample compartment, respectively.

## 4. Results and Discussion

### 4.1. THz Spectroscopy Analysis

**Figure 3:**
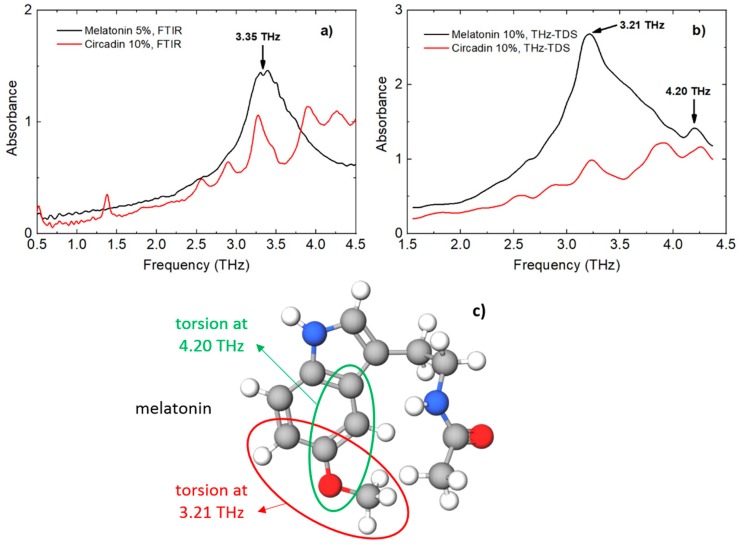
THz absorption spectra of melatonin and Circadin obtained by (**a**) FTIR and (**b**) THz-TDS. (**c**) Torsion modes of melatonin responsible for the spectral lines in the spectral region between 1.5 THz and 4.5 THz.

THz-TDS measurements for melatonin and Circadin were focused on a frequency range between 1.5 THz and 4.5 THz. The FTIR spectrum of melatonin does not exhibit any characteristic peaks below 1.5 THz, as shown in Figure 3a. In case of Circadin, the peak around 1.4 THz most likely belongs to lactose monohydrate as reported by other authors [50,51]. The spectral feature at around 3.3 THz in the FTIR spectra of melatonin is clearly observable. The same absorption line occurs in THz-TDS spectra in Figure 3b, where the pure melatonin mixed with reference PE shows a broad and strong absorption peak at around 3.21 THz. An additional low-intensity absorption peak can be seen at 4.20 THz. The same absorption line was observed by Singh et al. [10] in FTIR and also Raman experimental spectra of melatonin. The THz spectrum of Circadin pellet containing melatonin as API is also shown in Figure 3a. The predominant absorption peak of melatonin at 3.21 THz is visible in the THz spectrum of Circadin. In addition to this spectral feature, another absorption peak at 4.20 THz is observed as well. The characteristic absorption features for melatonin in the THz region below 5 THz can be assigned most probably to torsion modes, as reported by other authors [10]. In Figure 3c we encircled the regions around a methoxy group OCH_3_ within the melatonin molecule to which spectral features at 3.21 THz (red circle) and 4.20 THz (green circle) were assigned. According to the published simulated spectra of melatonin obtained by density functional theory (DFT) [10], we assume that bending, stretching, and wagging motions can be observed at higher THz frequencies that are beyond the scope of this work.

The appearance of the absorption peaks at three frequencies (2.65 THz, 2.90 THz, and 3.90 THz) of Circadin, which were not observed at the THz spectrum of pure melatonin, are most likely the contributions of characteristic peaks of lactose monohydrate, which is the predominant excipient in the Circadin tablet. These spectral features of lactose monohydrate agree with the measured and already published data [50,51,52]. In addition, the absorption peak of melatonin at 3.21 THz is probably shifted to the higher frequency due to the overlapping with the spectral feature of lactose monohydrate at 3.45 THz. The absorption peak of melatonin in Circadin at 4.20 THz can also be distorted due to several spectral lines of lactose monohydrate that are present in the frequency range from 4.0–4.5 THz [50]. The spectral features of melatonin and lactose monohydrate obtained experimentally with THz-TDS and FTIR, as well as the published spectral data, are summarized in Table 2. From these results, it can be concluded that the spectral contributions from melatonin are observed in the Circadin sample. An appropriate approach of data analysis may allow the characteristic spectral lines of the melatonin to be distinguished from the spectral features of other excipients present in Circadin. 

**Figure 4:**
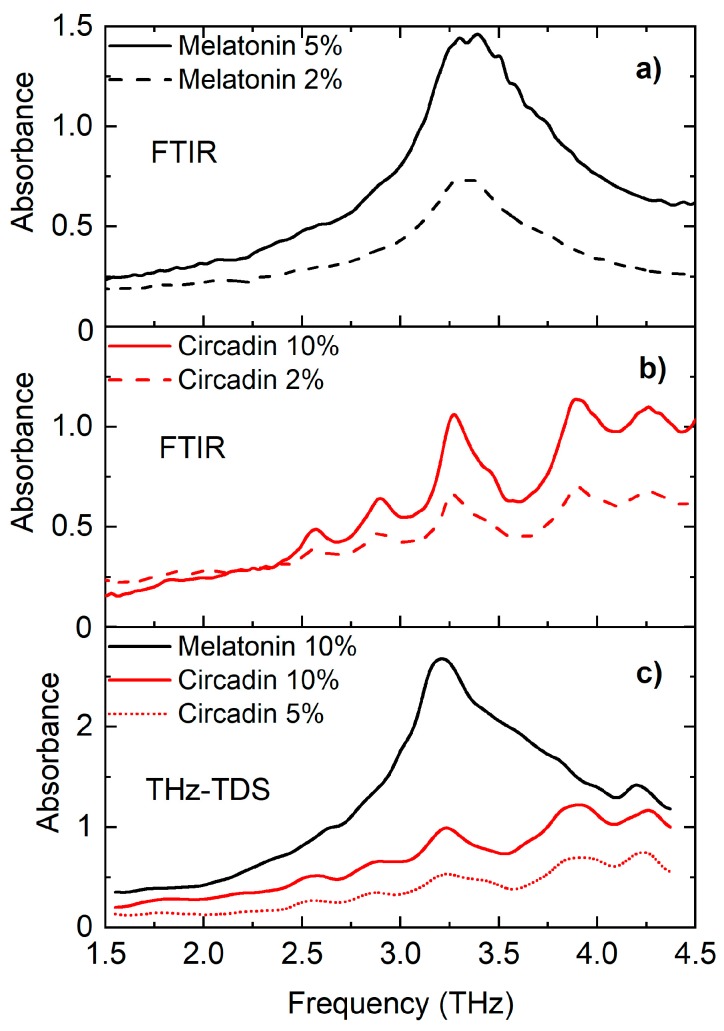
Concentration-dependent THz absorption spectra of (**a**) melatonin obtained by FTIR, (**b**) Circadin obtained by FTIR melatonin, and (**c**) melatonin and Circadin obtained by THz-TDS.

Figure 4a shows concentration-dependent THz absorption spectra in the frequency range 1.5–4.5 THz of melatonin obtained by FTIR spectrometer at room temperature. For two samples with various concentrations of melatonin (2 wt% and 5 wt%), a common absorption peak at around 3.20 THz was observed, which dominated in both spectra. In addition, the comparison of the FTIR spectra of melatonin with two different concentrations shows that the absorbance increased with the increased concentration of melatonin. Figure 4b shows a comparison of the THz spectra of Circadin with two different concentrations within the PE matrix. Several absorption peaks in both spectra are observable. Of these, two belonging to melatonin might also be assigned to lactose monohydrate as reported by other authors (Table 2). Figure 4c shows THz spectra of melatonin and Circadin with two concentrations obtained by THz-TDS. The absorption peaks of melatonin coincide with the absorption lines of Circadin for both concentrations. As it can be seen, the THz spectroscopy is able to identify different concentrations of the melatonin in a mixed form, and thus allows for a qualitative and quantitative analysis of APIs like melatonin within pharmaceutical products.

### 4.2. THz Imaging Analysis

Spectroscopic THz imaging is a convenient tool for security systems, allowing us to distinguish packaged materials if their spectra are known in advance [12,53]. Spectroscopic THz images can show not only the location of chemicals within the heterogeneous tablet but also identify each individual chemical compound within, as demonstrated by Kawase et al. [33]. Hereby, the THz spectroscopic imaging is used to identify melatonin-related pharmaceutical drugs as well as to detect its location within the pellet. The dimensions of the sample holder used for the acquisition of the images was 44 mm × 27 mm. First, THz pulse transmission through the sample was measured pixel-by-pixel in the time domain. In total, 1188 waveforms were recorded, each of them containing 360 points, an equivalent to a temporal scan length of 10 ps. For each waveform, the Fourier transform spectrum was calculated, as well as the absorption spectrum by computing the negative logarithm of the observed image intensity divided by the reference illumination THz intensity. Next a three-dimensional (3D) matrix data set was constructed, where two axes described the horizontal and vertical dimensions, while the third axis described the spectral frequency dimension. The spectral range of the measurements in the 3D matrix was set from 1.5 THz to 4.5 THz, according to the above described THz spectroscopic analysis. 

**Figure 5:**
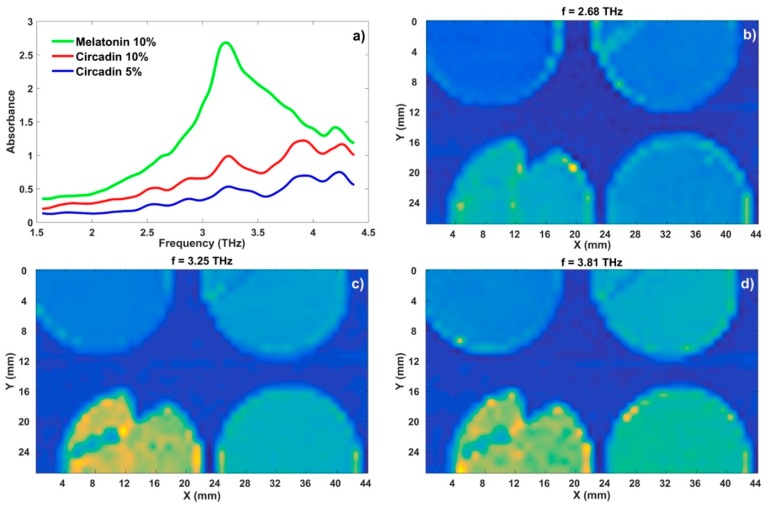
(**a**) THz spectra of melatonin and Circadin with two different concentrations. (**b**–**d**) THz spectroscopic images at the most representative discrete frequencies. On each THz image the samples/pellets are arranged as follows: Top left—PE, top right—Circadin 5 wt%, bottom left—melatonin 10 wt%, bottom right—Circadin 10 wt%.

Figure 5 shows THz spectroscopic images at the most representative discrete frequencies obtained from a 3D absorption matrix data set at 2.68 THz, 3.25 THz, and 3.81 THz. Images were interpolated by a factor of 4 to improve the resolution. The yellow color represents the maximum absorption at the selected frequency, whereas the dark blue color indicates a region with low THz absorption. Note that there is no significant absorption at 2.68 THz in pellets as the samples are almost transparent to these THz waves, hence blue color dominates for all three samples. The absorption of melatonin pellets increases up to ~3.25 THz, where the characteristic peak is seen in Figure 5a (green curve). In the case of Circadin, the absorption at this frequency is less expressed, since the Circadin with melatonin and other constituents is more transparent than the pure melatonin of the same concentration in the PE pellets. Above the characteristic peak of melatonin, the absorption of melatonin pellet decreases, whereas in Circadin pellets the THz spectra have an intense spectral feature around 3.90 THz (red and blue curves in Figure 5a), which likely belongs to lactose monohydrate. Therefore, the absorption intensity of Circadin pellets is close to the absorption of melatonin pellet at this frequency, and thus yellow shades can also be observed in the Circadin pellets (Figure 5d).

These results show that THz spectroscopy and THz spectroscopic imaging can become an indispensable analytical tool in the recognition and identification of API in pharmaceutical products and counterfeits, thus complementing other known methods such as FTIR, NMR, vibrational spectroscopy, Raman spectroscopy, nuclear quadrupole spectroscopy, chromatography, and mass spectroscopy [20,54,55,56]. The spectroscopic THz approach is capable of detecting very small quantities, so that for substances with high absorption in THz range and signal post-processing algorithms, traces even below 1 mg could be identified. Combined with the simple system setup, this offers a practical tool in the analysis of pharmaceuticals and chemicals.

## 5. Conclusions

Terahertz spectra of melatonin and its pharmaceutical product Circadin have been measured in this work for the first time by using THz spectroscopy in the spectral range of 1.5–4.5 THz. Two characteristic spectral features were found, a predominant feature at 3.21 THz and a weaker one at 4.20 THz. The characteristic THz spectral feature of melatonin were also hereby verified with FTIR spectroscopy. We showed that THz spectroscopic imaging can be used to distinguish samples with different concentrations of an active pharmaceutical ingredient, in our case whole melatonin and Circadin pellets. Additionally, we demonstrated the feasibility of utilizing THz spectroscopy and spectroscopic imaging as a complementary method to FTIR in applications of identification and quality control in the pharmaceutical industry.

## Figures and Tables

**Table 1:** Pellets composition for THz-TDS measurements.

Sample	Polyethylene	Constituent 2
PE pellet	320 mg	NA
Low concentration melatonin pellet	304 mg	16 mg melatonin
High concentration melatonin pellet	288 mg	32 mg melatonin
Low concentration Circadin pellet	304 mg	16 mg Circadin (0.1 mg melatonin)
High concentration Circadin pellet	288 mg	32 mg Circadin (0.2 mg melatonin)

**Table 2:** Absorption peaks for melatonin and lactose monohydrate in frequency range 1.0–4.5 THz obtained by THz-TDS and FTIR in this research and summarized from other sources.

Chemical Compound	Absorption PeakTHz-TDS (This Work)		Absorption Peak FTIR (This Work)		Absorption PeakFTIR (Published 1–3)	
	THz	cm^−1^	THz	cm^−1^	THz	cm^−1^
**Melatonin**	3.21	107	3.35	112	3.60 ^1^	120
	4.20	140			4.11 ^1^	137
**Lactose Monohydrate**					1.42 ^2,3^	47
					1.85 ^3^	62
					2.65 ^3^	88
					2.95 ^3^	98
					3.45 ^3^	115
					3.90 ^3^	130
					4.0 ^3^	133
					4.2 ^3^	140
**Circadin (melatonin)**	3.25	108	3.25	108		
	4.25	142	4.25	142		
**Circadin (lactose monohydrate)**			1.40			
	2.65	88	2.65	88		
	2.90	97	2.90	97		
	3.90	130	3.90	130		

^1^ summarized from [10], ^2^ summarized from [47], ^3^ summarized from [50].

## Supplementary Materials

Supplementary File 1
