# Supplementary material for: Spectroscopic Analysis of Melatonin in the Terahertz Frequency Range
Source: Sensors (Basel). 2018 Nov 23;18(12):4098. doi: 10.3390/s18124098 (PMC6308847; doi:10.3390/s18124098)
Supplement: Supplementary File 1 [file sensors-18-04098-s001.pdf]

Supplementary material

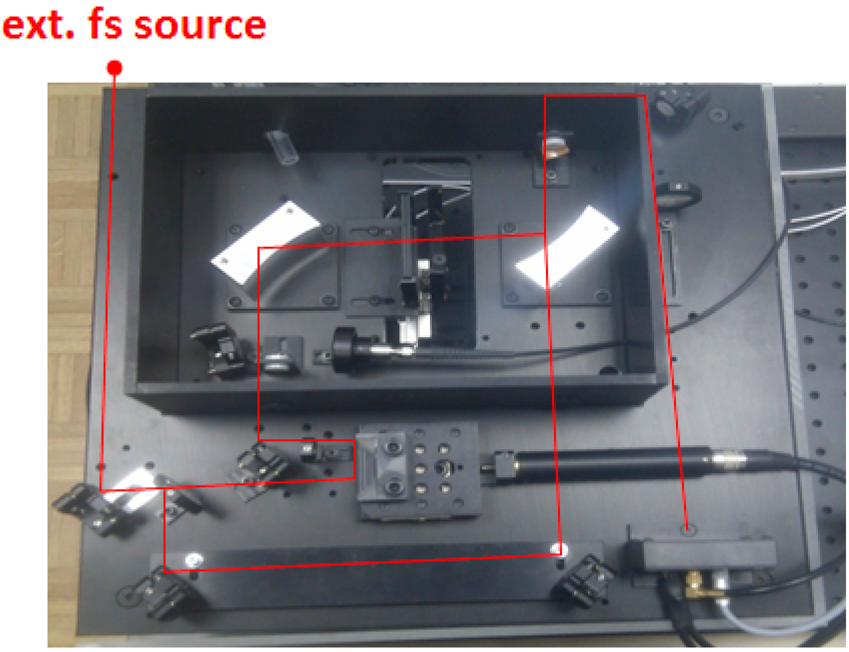

Figure S1: Picture shows the main part of the THz-TDS system used in this work. At the left side of the picture, the fs laser beam enters the THz-TDS system and passes through the beam splitter where is divided into the pump and probe beam. The pump beam goes through the delay line and hits the DSTMS crystal inside the sample compartment where THz-waves are generated. The THz radiation is then reflected from the first mirror, passes through the sample, and is finally reflected by the second mirror to the THz detector crystal. At the same place, the fs probe beam hits the detector crystal. The diode detector then detects changes in the probe beam.

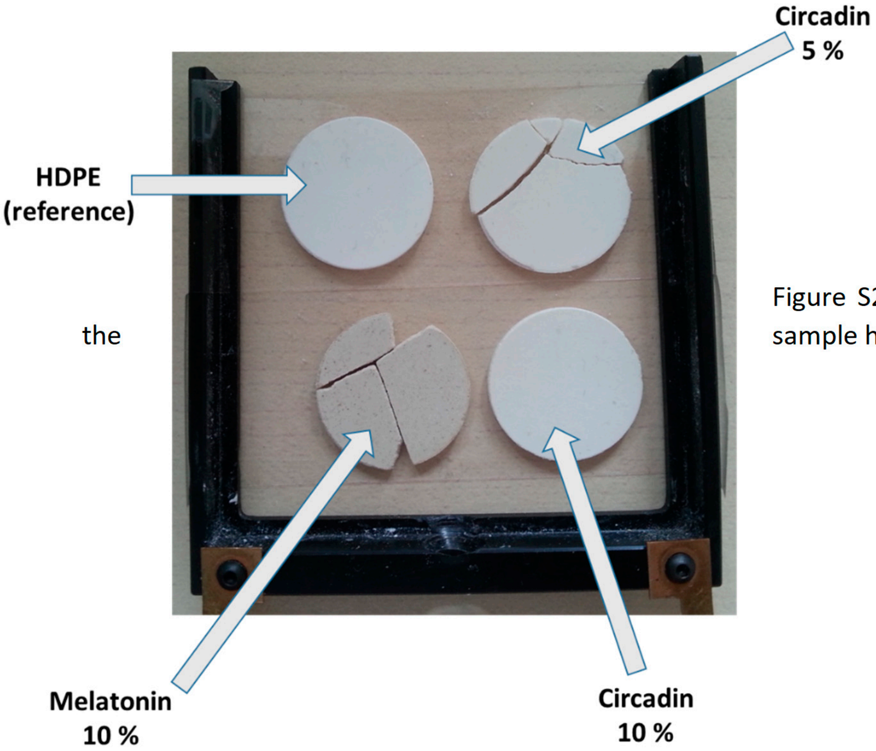

Figure S2: Photo of the samples placed in the sample holder.
